# Hypersensitivity Pneumonitis: An Interesting Case of Acute Shortness of Breath in a Young Patient

**DOI:** 10.7759/cureus.68683

**Published:** 2024-09-05

**Authors:** Mubashir Rafique, Faisal Arslan, Joohi Khan, Sameh Zaki, Ali Hussain

**Affiliations:** 1 Cardiology, Pinderfields Hospital, Wakefield, GBR; 2 Acute Medicine, Pinderfields Hospital, Wakefield, GBR; 3 General Practice, Pinderfields Hospital, Wakefield, GBR

**Keywords:** bird fancier disease, ild interstitial lung disease, exertional dyspnea, avian protein, hypersensitivity pneumonitis (hp)

## Abstract

Hypersensitivity pneumonitis (HP) is a rare disease caused by an inflammation of the distal airway caused by an immune response to inhaled allergens. The clinical presentation and radiological and histological findings can overlap with other pulmonary conditions such as idiopathic pulmonary fibrosis. Therefore, it is essential to consider focused assessment for the patient if a diagnosis of HP is suspected. We present a case involving a young female patient who presented with symptoms of cough, flu-like illness, and dyspnea. Subsequent investigations revealed a diagnosis of nonfibrotic HP. The patient experienced acute respiratory failure and was managed with high-flow oxygen therapy. A detailed investigation determined that the patient's prior exposure to pet parrots at home was a significant factor. Following treatment with steroids and counseling regarding the removal of parrots from the home environment, the patient's condition improved, and she was successfully weaned off of oxygen therapy. This case underscores the importance of a comprehensive social history in evaluating common complaints such as dyspnea. The rarity of parrot-induced HP related to the patient's age, and exposure warrants attention.

## Introduction

Hypersensitivity pneumonitis (HP) is a condition wherein the immune system responds to inhaled allergens, leading to inflammation in the distal airway [1]. This can be classified as nonfibrotic and fibrotic based on symptom duration and high-resolution CT (HRCT) findings. Acute HP is characterized as acute/inflammatory symptoms lasting less than six months, whereas chronic/fibrotic (fibrotic alterations on HRCT indicate symptoms that last longer than six months) [2]. This is typically presented as shortness of breath and cough with audible rales on chest examination [3]. The typical findings include centrilobular ground glass nodules, mosaic attenuation, and air trapping. The most specific findings are three density signs (head cheese sign) and exposure to known causes of HP (e.g., history of exposure to avian protein). Lymphocytosis in bronchoalveolar lavage and histological findings, suggesting HP [4]. There is no standardized management for HP, but the avoidance of exposure and immunosuppressants in the form of steroids form the basis for treating HP, especially the acute/inflammatory forms of HP [5].

## Case presentation

A 19-year-old female university student with no background cardiac or respiratory disease presented in ED with acute onset progressive shortness of breath. Her symptoms started with a dry cough, but she denies any history of hemoptysis or fever. She trialed a course of inhaled bronchodilators with no significant benefit. She developed orthopnea, used five pillows overnight, and had paroxysmal nocturnal dyspnea. Moreover, she also had exertional dyspnea and chest tightness. She had been vaping for two years; however, she stopped vaping after these symptoms. She used to drink alcohol and never used any recreational drugs.

On arrival at the ED, she had low oxygen saturation and was tachypneic. She was started on oxygen by a facemask. Her blood pressure, heart rate, and temperature were unremarkable. Her chest examination revealed bilateral fine rales. The jugular venous pulse was not elevated, and there was no clinical evidence of oedema or deep vein thrombosis on the leg examination. The rest of the physical examination was unremarkable. COVID-19 and respiratory viral panels were negative. Her baseline investigations (Table 1), were unremarkable. Troponin and procalcitonin levels were normal. The ECG was negative for evidence of ischemia, and the echocardiogram did not show evidence of heart failure or pulmonary hypertension.

**Table 1:** Baseline laboratory parameters. anti-CCP (anti-cyclic citrullinated peptides); IU: international units

Entity	Results	Normal values with units
Hemoglobin	153	130-180 g/L
White blood cells	8.9	4.0-11.0 109/L
C-reactive protein (CRP)	36	0-9 mg/L
Sodium	137	133-146 mmol/L
Potassium	4.6	3.5-5.3 mmol/L
Urea	3.7	2.5-7.8 mmol/L
Creatinine	68	45-84 µmol/L
Alanine aminotransferase (ALT)	11	0-56 µ/L
Alkaline phosphatase (ALP)	126	30-130 U/L
Bilirubin	16	0-21 µmol/L
Albumin	42	35-50 gm/L
D-dimer	0.75	Normal
Troponin T	<3	0-10 ng/L
Creatinine kinase (CK)	41	25-200 U/L
Rheumatoid factor	<10	0-14 IU/mL
Procalcitonin	<0.02	<0.05 ng/mL
Anti-citrulline antibody (anti-CCP)	2.4	0-6.9 U/mL

**Figure 1:**
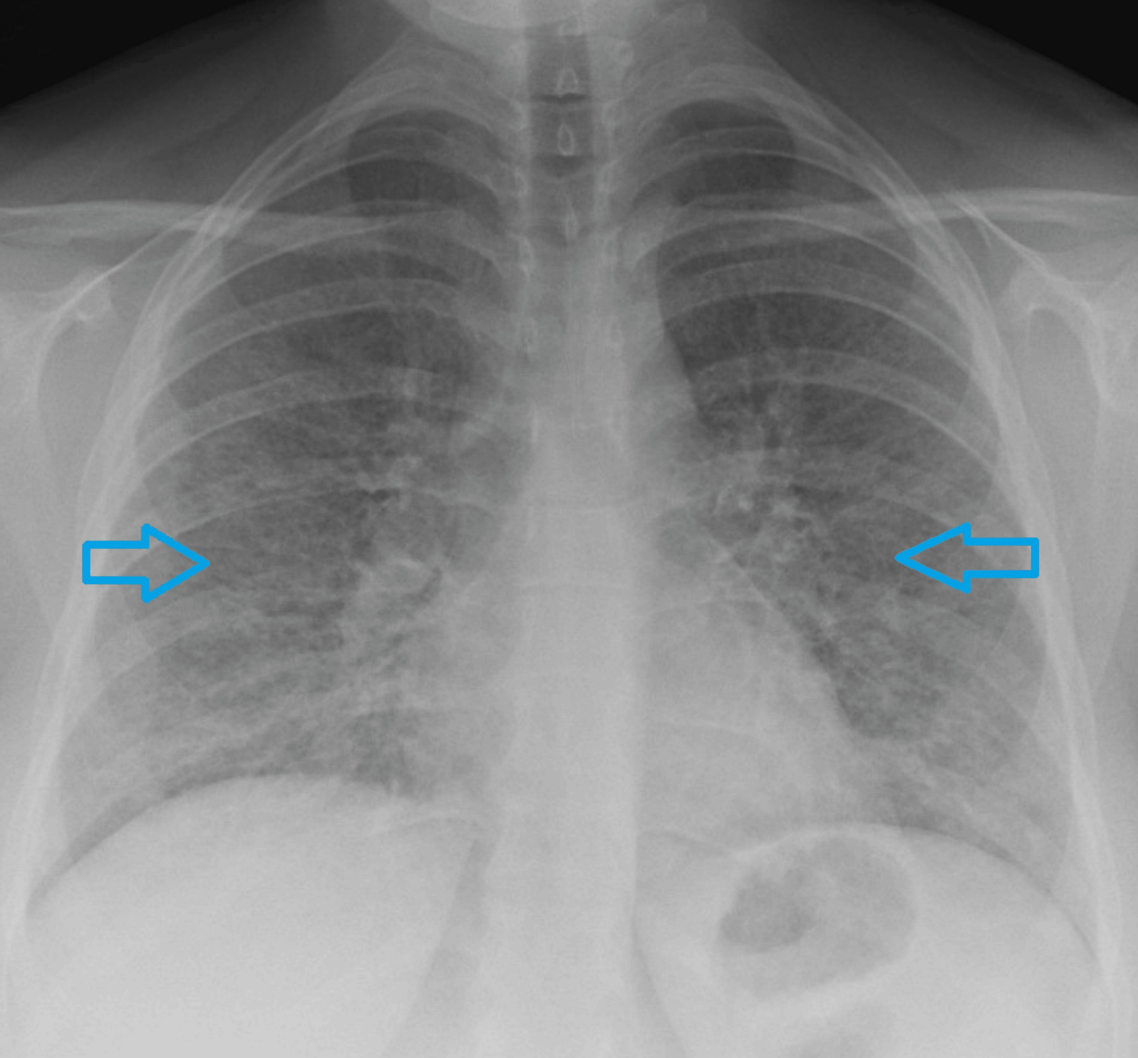
Bilateral reticular interstitial shadowing as shown by arrows.

A chest X-ray (Figure 1) showed increased bilateral reticular shadowing with apparent peri bronchial cuffing with no identified pleural effusion.

**Figure 2:**
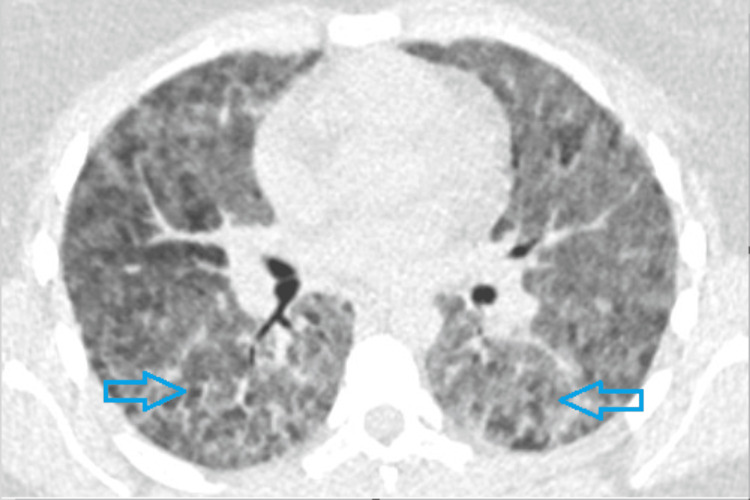
Bilateral symmetrical ground-glass changes in both lungs (as shown by arrows), which is in line with hypersensitivity pneumonitis.

HRCT (Figure 2) indicated bilateral symmetrical ground-glass change throughout both lungs, keeping with acute to subacute HP.

Her vasculitis screen, myositis immunoblots, and HIV test were normal, ruling out these potential causes of her symptoms. The total IgE levels were 139 kU/L (< 100). Detailed viral throat polymerase chain reaction (PCR) swabs were negative.

Her overall clinical presentation suggested HP. Therefore, detailed occupational and social history was explored. She lived in a well-ventilated house. She has had two parrots (Macaw and African Grey) for a few years. She enjoyed Viking reenactment with martial arts as her hobby. After confirmation of bird exposure, an avian protein panel was ordered, which was strongly positive, as shown in Table 2.

**Table 2:** Antibody panel for avian protein.

Test	Result	Reference range
Avian protein IgG budgie	154.0 mg/L	Value > 7.9 mg/L is likely to indicate a significant reaction to budgie antigen
Avian protein IgG pigeon	58.2 mg/L	Value > 9.9 mg/L is likely to indicate a significant reaction to bird antigen

**Figure 3:**
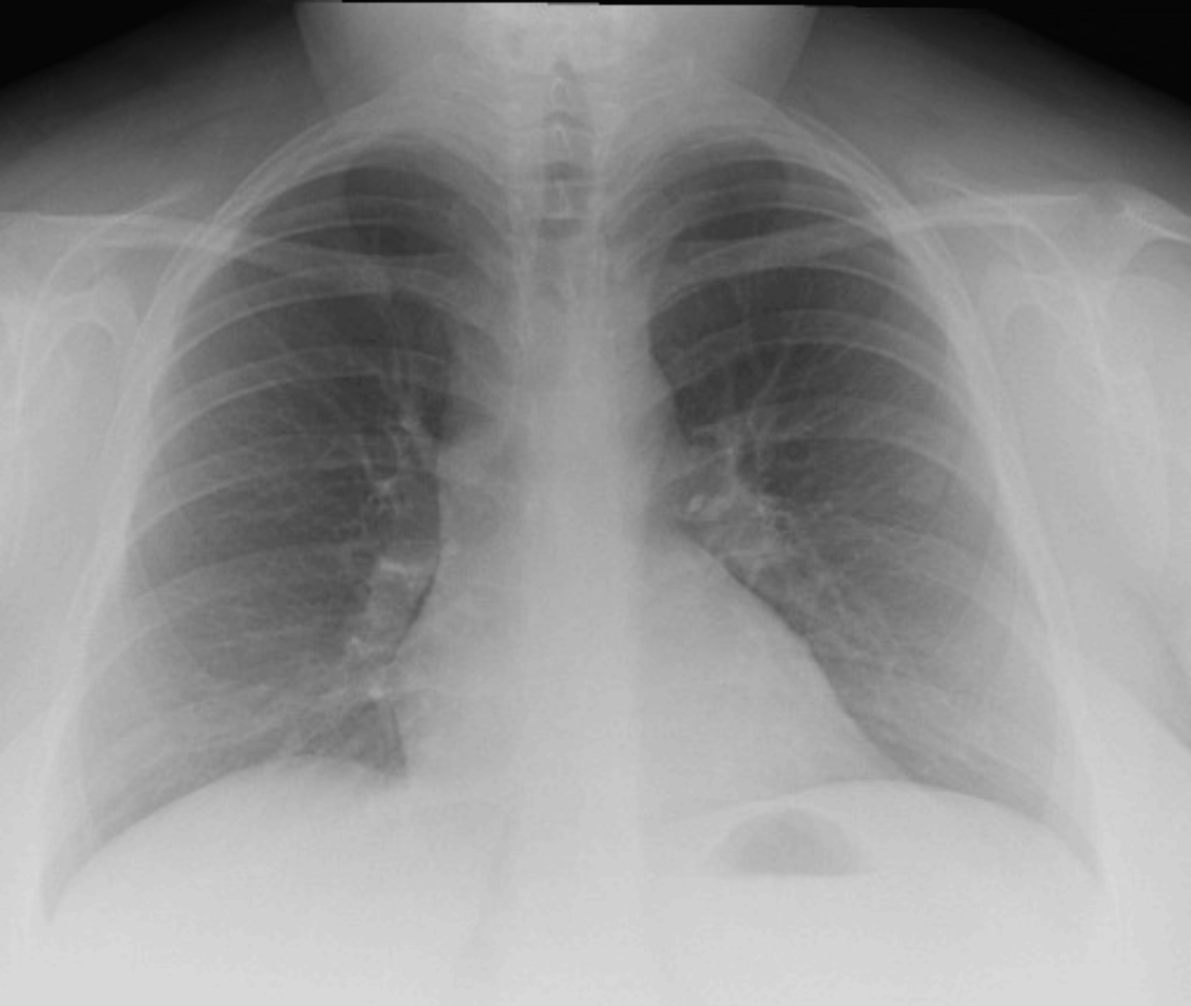
Follow-up X-ray after treatment shows complete resolution of reticular interstitial shadowing.

She responded well to oral steroids and subsequently weaned off of oxygen. She was discharged home with maintenance steroids. She was counseled to shift to a new home amid an occupational team visit to clear the house of avian protein and avoid contact with pigeons. She has been regularly followed up with a chest clinic with subsequent improvement in her symptoms and chest X-ray (Figure 3).

## Discussion

HP is an immune-mediated inflammatory and fibrotic disease that manifests as interstitial lung disease (ILD) after exposure to an identified or unidentified antigen [1]. The most common causative antigens for HP are bird and avian protein exposure, leading to forms named “bird fancier’s” and “pigeon breeder disease” [6]. The typical sources for animal proteins causing HP include avian droppings from parakeets, canaries, budgerigars, pigeons, parrots, chicken, turkeys, geese, ducks, wild birds, pheasants, and avian feathers from feather beds, pillows, and duvets [1]. The estimated incidence of HP is between 0.3 and 0.9 per 100,000 individuals, but the data have not been standardized because of regional disparities [7].

It is fascinating why only a tiny percentage of people get HP, considering the widespread and universal distribution of the causing antigens. There has been talk of a "two-hit" theory, according to which exposure to antigen (the second hit) raises the chance of developing HP if there is prior genetic vulnerability or environmental variables (the first hit). Genetic and environmental factors promote risk, while antigen exposure is the initiating factor [8].

Based on the presentation, HP has been classified as acute, subacute, and chronic. However, more recently, an objective classification by the American Thoracic Society (ATS), European Respiratory Society (ERS), Japanese Respiratory Society (JRS), and Latin American Thoracic Society (ALAT) recommended that patients be categorized as either nonfibrotic HP or fibrotic HP, depending on whether radiological or histological fibrosis is more prevalent [1]. Another classification has grouped HP as acute/inflammatory (symptom duration usually less than six months) and chronic/ fibrotic HP (the presence of fibrotic changes on HRCT typically correlating to symptoms more than six months) [2].

During chest examination, HP typically presents with dyspnea, cough, mid-inspiratory squeaks, and sometimes associated constitutional symptoms with audible rales [3].

The diagnosis for HP can be made by a multidisciplinary discussion of the relevant clinical history of significant exposure, radiological investigation such as HRCT, pathological examination of lung parenchyma, and immunological studies such as immunoglobulin (IgG) for suspected antigen and bronchoalveolar lavage [4].

Acute presentations and nonfibrotic disease are typically marked by patchy, nodular infiltrates on the chest X-ray, with fibrosis and peribronchial thickening appearing in later stages [9]. However, a chest radiograph (CXR) is nonspecific, and HRCT is almost always required.

HRCT can also indicate the usual information used to categorize the disease. Upper and middle lobe ground-glass attenuation, centrilobular nodules, and mosaic attenuation air trapping are consistent with acute HP. In contrast, upper and mid-zone fibrosis, peri-broncho vascular fibrosis, and honeycombing suggest chronic HP [10]. The three-density pattern, formerly known as the head-cheese sign, is a patchy distribution of normal-appearing lobules, ground glass, and lobules with decreased lung density and vascular size. This is the CT pattern with the highest specificity for HP [11]. Diagnostic uncertainty remains, as some radiological findings, such as mosaicism or air trapping, are also seen in idiopathic pulmonary fibrosis [12].

Pulmonary function tests (PFTs) are typically abnormal, demonstrating either restriction or obstruction or both, and the diffusing lung capacity of carbon monoxide (DLCO)is reduced. PFTs and DLCO may be expected between acute attacks.

Bronchoalveolar lavage (BAL) is sometimes necessary to exclude another differential diagnosis. Typically, in patients with HP, there is lymphocytosis [13]. Although there is no consensus on the diagnostic criteria for HP, the detailed social history exploring the patient's exposure to the triggers is paramount, and it can prevent them from undergoing unnecessary investigations.

The main diagnostic challenge is differentiating HP from other causes of shortness of breath and overlapping presentation. COVID-19 and acute heart failure can present in a similar pattern. COVID-19 PCR is negative in HP, and the clinical presentation of HP lacks features of heart failure, such as fluid overload, normal B-type natriuretic peptide (BNP), and echocardiogram, as in our case. Acute respiratory distress syndrome (ARDS) can mimic radiologically HP; in ARDS, CXR shows bilateral coalescent opacities, whereas for numerous small opacities <5 mm, ground glass appearance and fine reticulation are observed in HP. BAL may help differentiate HP from other causes of ILD. There is no extrapulmonary fibrosis in HP as compared to sarcoidosis. BAL shows lymphocytes in HP, which helps differentiate it from idiopathic pulmonary fibrosis and desquamative interstitial pneumonia. Conversely, in pneumoconiosis (associated pleural plaques and hilar lymph nodes egg cell calcification) and organic toxic dust syndrome, BAL shows increased neutrophils rather than lymphocytes.

There is no widely available therapeutic regimen for the management of HP. The most effective treatment for HP is identifying the trigger and avoiding further exposure. This may involve workplace reassignment, environmental interventions, and hygiene, such as dehumidifiers and adequate/well-maintained ventilation systems. These interventions are more effective during the acute phase of HP and less valuable during the chronic form of HP [14]. The time it takes for symptoms to reduce depends on the patient, antigen, and environment. Pulmonary rehabilitation therapy is beneficial for the patient's quality of life and functional status. The 2020 American Thoracic Society guidelines recommend that supplemental oxygen should be administered in cases of severe hypoxemia (PaO₂ (partial pressure of oxygen) ≤55 mmHg or oxygen saturation ≤89%), whether at rest or during effort. Immunosuppression in the form of corticosteroids is a mainstay treatment, although data are lacking [5]. The recommended dose for prednisolone is 0.5 to 1 mg/kg/day orally, taper dose of 5-10 mg/day every other day for six weeks. Long-term corticosteroid treatment is used for chronic fibrotic HP, although the role is not beneficial. Pulmonary function testing is likely the best way to monitor prednisolone tapering. Other immunosuppressants such as mycophenolate mofetil (MMF) and azathioprine (AZA) have shown significant improvement in carbon monoxide transfer factor (TLCO); however, they have no benefit for survival [15]. Lung transplant improves survival in selected patients with fibrotic HP. According to the International Society of Heart and Lung Transplantation, individuals with HP should be referred for lung transplant evaluation soon to increase their chances of being eligible for listing [16].

Complications for HP include progressive fibrosis leading to the deterioration of pulmonary function, pulmonary hypertension, hypoxemia, and ultimately death. The prognosis for individuals with fibrotic HP is determined by lung function at diagnosis, the amount of fibrosis that has already developed, and avoidance of antigens. Pulmonary function tests are unlikely to return to normal if there is notable fibrosis. Older age, male sex, cigarette smoking, a reduced baseline vital capacity, the absence of bronchoalveolar lavage lymphocytosis, continuous exposure to the causative antigen, and the inability to identify a causative antigen are additional characteristics linked to an undesirable prognosis [17].

## Conclusions

HP is a complex and challenging diagnosis; the clinical presentation and radiological and histological findings can overlap with other cardiac and pulmonary conditions. Detailed occupational and exposure history is helpful in clinching diagnosis. Avoidance of further exposure to avian protein can halt the progression of the disease. Complications for HP include progressive fibrosis leading to deterioration of pulmonary function, pulmonary hypertension, hypoxemia, and, ultimately, death. These can be prevented by promptly diagnosing the disease, educating the patient on how to minimize contact with birds, and close regular follow up with the clinician.
